# Survivin, Ki-67 and tumor grade as predictors of response to docetaxel-based neoadjuvant chemotherapy in locally advanced breast cancer

**DOI:** 10.3892/mco.2013.138

**Published:** 2013-06-18

**Authors:** QI LIN, YANG LIU, HUIYU CHEN, YI LIU, QIANG TANG, JING LIU, HAO CHEN

**Affiliations:** 1 Department of General Surgery, Zhongshan Hospital Affiliated to Fudan University, Shanghai 200032, P.R. China; 2 Department of Ultrasonography, Lianyungang First People's Hospital Affiliated to Xuzhou Medical College, Lianyungang, Jiangsu 222002, P.R. China; 3 Department of General Surgery, Lianyungang First People's Hospital Affiliated to Xuzhou Medical College, Lianyungang, Jiangsu 222002, P.R. China; 4 Department of Pathology, Lianyungang First People's Hospital Affiliated to Xuzhou Medical College, Lianyungang, Jiangsu 222002, P.R. China

**Keywords:** breast cancer, neoadjuvant chemotherapy, predictive factors, docetaxel-based chemotherapy regimen

## Abstract

The response rates to neoadjuvant chemotherapy (NAC) in patients with locally advanced breast cancer (LABC) may vary and the risks may outweigh the benefits in poorly selected patients. This study investigated whether survivin expression, high-level Ki-67 expression, estrogen-receptor (ER) tumor status and high tumor grade are able to predict response to docetaxel-based NAC in LABC patients, in order to perform breast-conserving surgery. In this study, 68 patients (IIb-IIIb) completed 4–6 cycles of TAC (75 mg/m^2^ docetaxel, 60 mg/m^2^ pirarubicin and 500 mg/m^2^ cyclophosphamide, administered every 3 weeks). Tumor samples were obtained prior to chemotherapy. The response to chemotherapy was quantified clinically and pathologically and the histological and molecular tumor characteristics were determined. Association with response was assessed for all the parameters and the patients underwent breast-conserving surgery or radical mastectomy accordingly. A clinical complete response was observed in 21 (31%) and a partial response in 37 (54%) of the 68 patients. Thus, the overall clinical response rate (ORR) was 85%. A pathological complete response (pCR) was observed in 14 (20%) of the 68 patients and 37 patients (54%) underwent breast-conserving surgery. In the univariate analysis, survivin expression, high-level Ki-67 expression and high tumor grade (grade III) were significantly associated with ORR (P=0.007, 0.024 and 0.047, respectively). Survivin expression and high-level Ki-67 expression were significantly associated with pCR (P=0.029 and 0.048, respectively). In the multivariate analysis, survivin expression (P=0.030) and tumor grade (P=0.036), but not high-level Ki-67 and ER expression, were significantly associated with ORR and none of these factors was significantly associated with pCR. In conclusion, expression of survivin and high tumor grade were of predictive value for ORR to docetaxel-based NAC in LABC patients, leading to more patients successfully undergoing breast conserving-surgery. Immunohistochemistry of survivin and the Elston and Ellis criteria of tumor grade may provide a widely applicable, cost-effective method of patient selection for NAC.

## Introduction

The response rates to neoadjuvant chemotherapy (NAC) in patients with locally advanced breast cancer (LABC) are 40–60%. The drawbacks of this treatment method are that certain patients may experience adverse effects and that it may cause unnecessary delay to surgical treatment, particularly in the cases with progressive disease. Therefore, biomarkers that predict response to NAC may prove useful. The most important qualities for a biomarker are that it may be assessed easily and at low cost, even in hospitals that lack expensive laboratory equipment and advanced molecular techniques. Previous studies demonstrated that gene microarray technologies are able to predict response to NAC (1,2). However, this advanced technique has not been routinely applied in the clinical setting due to its high cost, particularly in developing countries and low-income areas.

An increasing number of studies investigate factors [including survivin, Ki-67, estrogen-receptor (ER) tumor status and tumor grade] that may predict response to either first- or second-line chemotherapy. However, available data on the prediction of the efficacy of third-line chemotherapeutic agents (i.e., those including anthracyclines and either docetaxel or dose-dense weekly paclitaxel) are limited. The aim of this study was to investigate whether survivin, Ki-67, ER tumor status and tumor grade are useful predictive biomarkers for the response of the primary tumor to NAC with a docetaxel-based regimen.

We considered a regimen of 75 mg/m^2^ docetaxel, 60 mg/m^2^ pirarubicin and 500 mg/m^2^ cyclophosphamide (TAC) once every 3 weeks to be an effective option for NAC in LABC, in order that more patients benefit from undergoing breast conserving-surgery following treatment. Survivin expression and high tumor grade were shown to be independent predictors of response.

## Materials and methods

### Patients

Between January, 2009 and December, 2012, 71 patients with locally advanced (stage IIb and IIIb) breast cancer were enrolled at Lianyungang First People's Hospital (Jiangu, China). Eligible patients had core needle biopsy-confirmed breast cancer, were previously untreated and had locally advanced tumors that were potentially operable, without evidence of distant metastasis (Table I). Three patients did not complete the chemotherapy scheme due to the development of leukopenia and asthenia.

This study was conducted in accordance with the ethics principles of the Declaration of Helsinki. The study methods were approved by the Institutional Review Board of Lianyungang First People's Hospital and patients provided written informed consent prior to enrollment.

### Treatment

Prior to treatment initiation, tumors were measured by magnetic resonance imaging (MRI). Patients received the TAC regime, administered every 3 weeks for 4–6 cycles. Patients were administered dexamethasone premedication (8 mg orally every 12 h, 6 times, starting the day prior to treatment initiation) to prevent docetaxel-related hypersensitivity and fluid retention. Primary prophylaxis with granulocyte colony-stimulating factor (G-CSF) was not permitted. However, in patients who developed episodes of febrile neutropenia or infection, administration of G-CSF was mandatory in the subsequent cycles. Approximately 2 weeks after NAC, the patients underwent either breast-conserving surgery or modified radical mastectomy. Following surgery, the patients received chemotherapy, radiotherapy, endocrine therapy or a combination of these treatments.

### Treatment response

The clinical treatment response was assessed using the Response Evaluation Criteria in Solid Tumors (RECIST) (3). The overall clinical response rate (ORR) was defined as the complete and partial responses combined. The pathological complete response (pCR) was assessed after surgical resection of the remaining tumor and nodes and was defined as the absence of tumor cells, absence of persistent *in situ* disease and negative axillary lymph nodes. All samples were assessed by two pathologists at Lianyungang First People's Hospital.

### Evaluation of survivin, Ki-67 and ER tumor status

Core biopsy specimens were fixed in 10% neutral-buffered formalin for 24 h prior to processing and embedded in paraffin wax blocks at the pathology laboratory in our hospital. Sections (3 mm) were cut from each block, mounted on positively-charged slides and stained with hematoxylin and eosin.

Prior to immunohistochemical analysis, the tissue sections were deparaffinized and rehydrated in graded alcohols. The slides were subjected to heat-induced epitope retrieval by immersion in 0.01 M boiling citrate buffer (pH 6) in a pressure cooker for 3 min, followed by a 20-min cooling period and overnight incubation with monoclonal antibody [rabbit monoclonal anti-survivin (1:100, cat no: Z2159; Reta Corporation, Deerfield Beach, FL, USA), rabbit monoclonal anti-Ki-67 (1:100, cat no: Z2031; Reta Corporation) and rabbit monoclonal anti-ER (1:200, cat no: Z2021RS; Reta Corporation)]. Negative and positive control slides were also prepared. Histological classification was performed according to the WHO criteria and tumor grading was performed according to the Elston and Ellis criteria (4).

**Figure 1:**
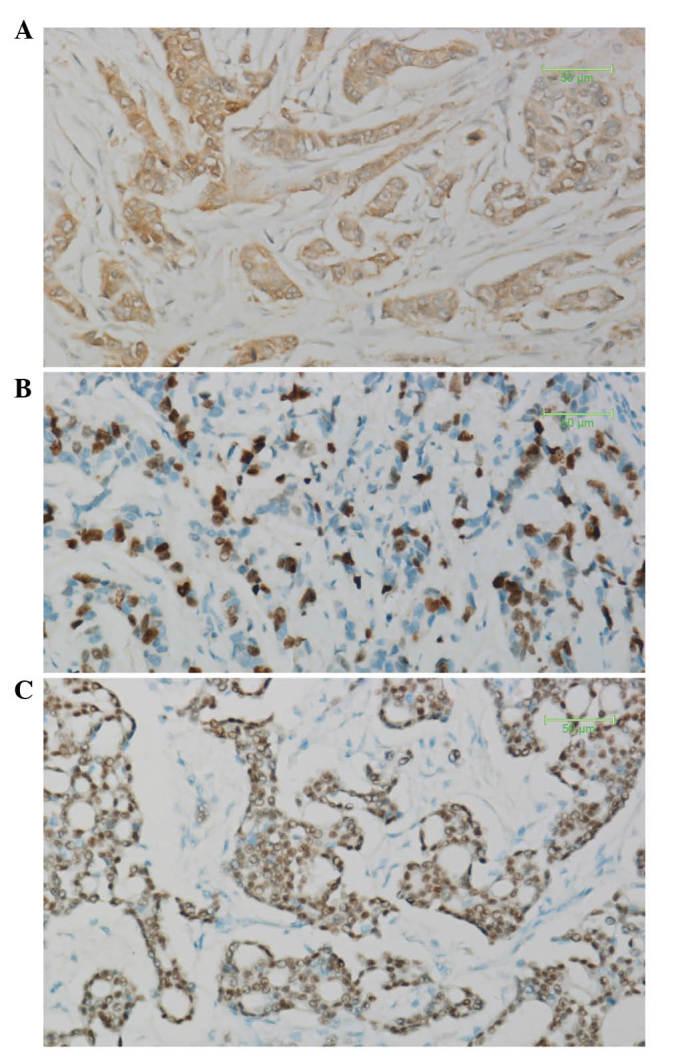
Immunohistochemistry results. (A) Survivin cytoplasmic expression. (B) Ki-67 nuclear expression. (C) Estrogen-receptor nuclear expression. Magnification, ×200.

Survivin expression was semi-quantitatively evaluated according to the percentage of cells with nuclear and/or cytoplasmic reactions. Immunoreactivity was assessed in at least five high-power fields at a magnification of ×200 and scores were classified as follows: 0, <5% of tumor cells stained; 1, 5–20% of tumor cells stained; and 2, >20% of tumor cells stained. A score of 2 was considered as positive and scores of 0 or 1 were considered negative (Fig. 1A) (5). For Ki-67, nuclear staining in >20% of the tumor cells was considered to indicate high-level expression (Fig. 1B). Tumors were classified as ER-positive when nuclear staining was visible in ≥10% of the tumor cells (Fig. 1C) (6).

### Statistical analysis

The primary endpoint was to assess the predictive value of survivin, Ki-67, ER-negative tumor status and tumor grade for the ORR to docetaxel-based NAC in patients with LABC, to enhance the breast-conserving surgery rate with docetaxel-based NAC. The effects of survivin, Ki-67, ER tumor status and tumor grade on the response to NAC and the correlations between survivin, Ki-67 and ER tumor status were assessed with the Pearson's Chi-square test (with a correction for continuity in comparisons with small numbers). Possible predictive factors associated with response probability at a significance level of ≤0.20 were considered in a multivariable logistic regression analysis (6). P<0.05 was determined as the threshold for statistical significance and all P-values were two-tailed. Data were analyzed with SPSS software for Windows version 16.0 (SPSS Inc., Chicago, IL, USA).

## Results

### Tumor response

MRI revealed that 21 (31%) of the 68 patients exhibited a clinical complete response and 37 (54%) exhibited a partial response. Therefore, the ORR was 85%. The disease was classified as stable in 11 (16.2%) of the 68 patients and no patient had progressive disease. pCR was confirmed in 14 (20%) of the 68 patients and 37 patients (54%) underwent breast-conserving surgery.

### Predictive value of survivin, Ki-67, ER status and tumor grade

Of the 68 breast carcinomas, survivin expression was detected in 50 (74%), with no expression observed in the adjacent normal tissue. High-level expression of Ki-67 was detected in 52 (77%) tumors and a ER-negative status was detected in 31 (46%) tumors. A total of 40 tumors (59%) were high-grade (grade III) and the remaining 28 (41%) were grade I/II.

In the univariate analysis, survivin expression, high-level Ki-67 expression and high tumor grade (grade III) were significantly associated with ORR (P=0.007, 0.024 and 0.047, respectively). Survivin expression and high-level Ki-67 expression were significantly associated with pCR (P=0.029 and 0.048, respectively) (Table I). In the multivariate analysis, survivin expression and high tumor grade, but not high-level Ki-67 expression, were significantly associated with ORR (P=0.030 and 0.036, respectively) and none of the factors was significantly associated with pCR (Table II).

### Correlation between biomarkers

A strong correlation was observed between the expression of survivin and the high-level expression of Ki-67 (P=0.034). It was not possible to clearly determine a correlation between survivin expression and ER-positive tumor status (P=0.223) or an inverse correlation between high-level Ki-67 expression and ER-positive tumor status (P=0.059).

## Discussion

Breast cancer is the most common cancer among women. NAC may lead to tumor downstaging and increase the likelihood of patients undergoing successful breast-conserving surgery. However, the benefits depend on the selection of the most effective chemotherapy regimens. Doctors commonly select regimens on the basis of clinical and histological characteristics and treatment is generally not individualized. Therefore, numerous patients may receive unnecessary or ineffective NAC, which may lead to toxic effects, increased cost, delay to curative treatment and tumor cross-resistance (7). Thus, methods to facilitate the selection of the most effective regimens are urgently needed.

Anthracycline-based regimens for breast cancer achieve high response rates. A widely used regimen for breast cancer NAC is combined cyclophosphamide, doxorubicin and fluorouracil; however, resistance to this regimen has emerged. Previous trials reported good response rates with taxane-containing regimens (8,9). Docetaxel has exhibited high activity in combination with doxorubicin, including in patients with anthracycline-resistant disease. Nabholtz *et al*(10) reported that patients with metastatic breast cancer treated with combined docetaxel and doxorubicin exhibited significantly improved ORRs compared to those of patients treated with doxorubicin and cyclophosphamide. Mackey *et al*(11) provided evidence in their 10-year analysis of the randomised BCIRG 001 trial that TAC for the treatment of patients with node-positive, early breast cancer provides long-term disease-free survival and overall survival benefits compared to fluorouracil (5-FU), doxorubicin and cyclophosphamide (FAC), irrespective of nodal, hormone receptor and human epidermal growth factor receptor-2 (HER2) status.

Despite the improvements in ORR and the assessment of several chemotherapeutic regimens, the pCR rate following NAC for breast cancer remains low (12). Survival is significantly prolonged in patients who achieve pCR after NAC compared to those who do not (13,14). This finding may, therefore, be used as a suitable surrogate endpoint for response in studies on NAC. In our study, after 4–6 cycles of the TAC regimen, the clinical CR rate was 31% and the pCR rate was 20%. Moreover, our primary endpoint was breast-conserving surgery. This suggests that the TAC regimen may be effective as NAC for patients with LABC.

The survivin gene (BIRC5) is a member of the inhibitor of apoptosis protein family and has various functions: regulation of cell proliferation and division, inhibition of cell apoptosis and promotion of angiogenesis (15). Survivin expression was detected by immunohistochemistry in 60–70% of primary breast tumors, with little or no expression in control tissue samples. High expression of survivin has been correlated with poor clinical outcomes in breast, lung, prostate, pancreatic and colorectal cancers (16). Survivin may, therefore, be a potential prognostic factor, a predictive factor for response to treatment and a therapeutic target in breast cancer patients. In the univariate analysis, we observed a significant correlation of survivin expression with ORR and pCR to NAC. However, in the multivariate analysis, a significant correlation was observed only with ORR. Several studies reported that patients with a pCR following NAC exhibited higher survival rates compared to those without pCR (13,14) and a high expression of survivin has been correlated with poor clinical outcomes (15,16). Therefore, the results of a previous study by Petrarca *et al*(18), indicating that survivin may be a predictive biomarker of pCR to NAC in patients with stage II and III breast cancer, were not in accordance with our results and require further investigation. Another study reported that failure of the downregulation of survivin following neoadjuvant radiochemotherapy in rectal cancer was associated with distant metastases and shortened survival (17), although there was no report of the association with breast cancer.

Gerdes *et al*(19) previously used a mouse monoclonal antibody against a nuclear antigen from a Hodgkin's lymphoma cell line and identified Ki-67 as a marker of cell proliferation. Ki-67 was universally expressed among proliferating cells and absent in quiescent cells. Several studies have since investigated the possible prognostic role for Ki-67 in breast cancer, with varying results. Urruticoechea *et al*(20) conducted a review of 40 studies involving >11,000 patients and reported that Ki-67 expression alone is able to predict positive or negative outcomes in patients with node-negative breast cancer, although the predictive value was not maintained in multivariate analyses. de Azambuja *et al*(21) demonstrated that Ki-67 expression in node-negative and node-positive breast cancer was associated with poor overall and disease-free survival. The findings of Stuart-Harris *et al*(22) did not support Ki-67 as a prognostic marker for use in routine practice. Sánchez-Muñoz *et al*(13) identified the Ki-67 index as an independent prognostic factor for disease-free and overall survival in breast cancer patients treated with NAC. A high Ki-67 expression and hormone receptor-negative status were shown to be predictors of pCR. In the univariate, but not the multivariate, analysis we observed that a high-level expression of Ki-67 was significantly associated with good ORR and pCR to NAC, which may be attributed to our small study sample. Although it is disputable whether Ki-67 is an independent predictor or prognostic marker, a high expression of Ki-67 indicating good response to docetaxel-based NAC suggests that breast-conserving surgery may be performed in the high-expression patients.

ER and progesterone receptor (PgR) status provides the index for sensitivity to endocrine treatment; therefore, it is the most important biomarker in breast cancer. Numerous studies identified ER and PgR as independent variables, significantly associated with the likelihood of achieving pCR (23,24). However, the findings of our study, taken together with those of Wang *et al*(25), do not support this result. This discordance may be explained by the heterogeneity of the investigation methods, particularly the cut-offs used by various studies. In our study, the sample size was small and the detection of PgR expression was performed after the NAC, which may change the status and affect the result. We observed that high histological grade was a significant independent predictor of pCR in multivariate models with low-grade tumors, which is consistent with the findings of previous studies in multivariate models (6).

A good correlation was observed between survivin expression and high-level Ki-67 expression, similar to the findings of Xu *et al*(26). A previous study by Lee *et al*(27) reported an inverse correlation between high-level Ki-67 expression and ER-positive tumor status. However, our study did not support that result and whether survivin expression correlates with ER-positive tumor status could not be determined. This was not in accordance with the findings of Ryan *et al*(28), possibly due to their detection methods for protein quantification being more accurate.

In conclusion, a regimen of 75 mg/m^2^ docetaxel, 60 mg/m^2^ pirarubicin and 500 mg/m^2^ cyclophosphamide once every 3 weeks may be effective as NAC in LABC and more patients may benefit from undergoing breast conserving-surgery after the treatment. Survivin expression and high tumor grade were identified as independent predictors of response.

## Figures and Tables

**Table I:** Univariate analysis of the association of baseline characteristics with ORR and pCR.

Characteristics	No.	ORR	P-value	pCR	P-value
No. of patients	68				
Median age, years (range)	45 (35–60)				
Tumor diameter, mm (range)	45 (25–80)				
Menopausal status					
Premenopausal	32	81% (26/32)	0.587^a^	19% (6/32)	0.724^a^
Postmenopausal	36	86% (31/36)		22% (8/36)	
ECOG performance					
0	44	86% (38/44)	0.670^b^	23% (10/44)	0.782^b^
1	24	79% (19/24)		17% (4/24)	
Clinical nodal status					
Negative	18	78% (14/18)	0.661^b^	17% (3/18)	0.889^b^
Positive	50	86% (43/50)		22% (11/50)	
Stage					
II (T≥5 cm)	30	80% (24/30)	0.668^b^	20% (6/30)	0.915^a^
III	38	87% (33/38)		21% (8/38)	
Survivin					
Negative	18	61% (11/18)	0.0072^c^	0% (0/18)	0.0292^a^
Positive	50	92% (46/50)		28 (14/50)	
Ki-67					
Negative	16	63% (10/16)	0.0242^c^	0% (0/16)	0.0482^a^
Positive	52	90% (47/52)		27% (14/52)	
Estrogen-receptor					
Negative	31	87% (27/31)	0.502^a^	29% (8/31)	0.330^a^
Positive	37	81% (30/37)		16% (6/37)	
No. of cycles					
4	39	85% (33/39)	1.0^b^	21% (8/39)	0.986^a^
5–6	29	83% (24/29)		21% (6/29)	
Tumor type					
Invasive ductal	60	87% (52/60)	0.218^b^	25% (13/60)	0.891^b^
Invasive lobular	8	63% (5/8)		13% (1/8)	
Tumor grade					
I/II	28	71% (20/28)	0.0472^c^	11% (3/28)	0.092^a^
III	40	93% (37/40)		28% (11/40)	
PgR					
Negative	37	28% (11/40)	0.189^a^	22% (8/37)	0.818^a^
Positive	31	77% (24/31)		19% (6/31)	
HER2 status					
0 to 1+	50	88% (44/50)	0.236^b^	24% (12/50)	0.412^b^
2+ to 3+	18	72% (13/18)		17% (2/18)	

a P, Pearson's Chi-square test,

b P, continuity correction test,

c P<0.05.

ORR, overall clinical response rate; pCR, pathological complete response; PgR, progesterone receptor. ECOG, Eastern cooperative oncology group; HER2, human epidermal growth factor receptor-2.

**Table II:** Multivariate analysis of the association of protein expressions with ORR and pCR.

Predictive markers	Odds ratio	95% CI	P-value
Survivin			
ORR	5.879	1.185–29.178	0.030^a^
pCR	3.652	0-0	0.998
Ki-67			
ORR	3.012	0.647–14.022	0.160
pCR	2.359	0-0	0.998
Tumor grade			
ORR	5.993	1.124–31.967	0.036^a^
pCR	0.254	0.537–10.474	0.254
PgR	0.460	0.091–2.328	0.348

a P<0.05.

CI, confidence interval; ORR, overall clinical response rate; pCR, pathological complete response; PgR, progesterone receptor.
